# Case Report: Telitacicept exposure in early pregnancy in a patient with SLE delivering an infant without any physical defect

**DOI:** 10.3389/fmed.2025.1737355

**Published:** 2026-01-21

**Authors:** Xiao-Shan Huang, Zhong-yu He, Shi-gang Wang, Qiang Xu, Chang-song Lin

**Affiliations:** 1 State Key Laboratory of Traditional Chinese Medicine Syndrome, The First Clinical Medical College of Guangzhou University of Chinese Medicine, Guangzhou, China; 2 Department of Rheumatology, The First Affiliated Hospital of Guangzhou University of Chinese Medicine, Guangzhou, China; 3 Guangdong Clinical Research Academy of Chinese Medicine, Guangzhou, China

**Keywords:** pregnancy, reproduction, safety, systemic lupus erythematosus, telitacicept

## Abstract

**Background:**

Systemic lupus erythematosus (SLE) is a systemic autoimmune disorder characterized by inflammatory damage to multiple organs. Its incidence rate is relatively high among women of childbearing age. The use of biologics is not recommended during pregnancy in SLE patients; belimumab or rituximab may be considered selectively only during lactation. Telitacicept is a fusion protein composed of the extracellular domain of the transmembrane activator and CAML interactor (TACI)—a receptor for B-lymphocyte stimulator (BLyS)—and the Fc fragment of human IgG1. It was first approved in China in 2021 for the treatment of patients with active SLE. Currently, there is a lack of safety data on the use of telitacicept during pregnancy.

**Case presentation:**

A 25-year-old woman with SLE was exposed to telitacicept during early pregnancy and delivered an infant without any physical defect. The patient was regularly receiving telitacicept injections. Her last dose was administered on 4 November 2024. Her last menstrual period was dated 7 November 2024, and telitacicept was discontinued upon pregnancy confirmation. During gestation, she was maintained on oral glucocorticoids, hydroxychloroquine, and tacrolimus. On 23 June 2025, at 32 weeks of gestation, she underwent a cesarean section due to “intrauterine growth restriction and preeclampsia” and delivered a male infant without any physical defects.

**Conclusion:**

This case suggests that exposure to telitacicept in the early stage of pregnancy did not result in structural defects in this case.

## 1 Introduction

Systemic lupus erythematosus (SLE) is a chronic, systemic autoimmune disease characterized by the production of autoantibodies, immune complex deposition, and multi-organ inflammatory damage. Its clinical manifestations are highly heterogeneous. It predominantly affects women with a female-to-male prevalence ratio of approximately 6:1. The global prevalence is about 5.14 per 100,000 people, and the peak incidence occurs between 15 and 45 years of age (1). Tissue damage is caused by abnormal activation of auto-reactive T and B cells and release of inflammatory cytokines (2).

The treatment of SLE is based on the combination of glucocorticoids and hydroxychloroquine. During the induction remission stage, immunosuppressants such as mycophenolate mofetil or cyclophosphamide may be added. For refractory cases, biologics such as belimumab or rituximab are used, along with comprehensive management including renin-angiotensin inhibitors and anticoagulants. SLE is more common in women of childbearing age, so the management of lupus during pregnancy is particularly important. It is recommended to plan pregnancy when the disease is stable (≥6 months) and there is no severe organ damage. The recommended drugs during pregnancy include hydroxychloroquine, low-dose aspirin (starting from the 12th week of pregnancy to prevent preeclampsia), glucocorticoids (prednisone ≤ 15 mg/d), azathioprine, cyclosporine, tacrolimus. During lactation, the above medications can be continued, and non-steroidal antiinflammatory drugs (NSAIDs) and biological agents such as rituximab, belimumab can be used as needed (3).

Approved in China in 2021 for the treatment of patients with active SLE, telitacicept is the world’s first dual-target three-channel biologic agent. Currently, successful cases have been reported regarding its use in refractory lupus erythematosus, immune thrombocytopenia, myasthenia gravis, IgA nephropathy, and granulomatosis with polyangiitis (4–9). However, no clinical studies have yet investigated the safety of telitacicept during pregnancy.

We report a case of a systemic lupus erythematosus patient exposed to telitacicept during early pregnancy delivering an infant without any physical defect.

## 2 Case presentation

This is the first reported case of a SLE patient who was exposed to telitacicept during early pregnancy and delivered an infant without physical defects. This finding suggests that telitacicept exposure during early pregnancy provides preliminary reassurance regarding teratogenicity.

**FIGURE 1:**
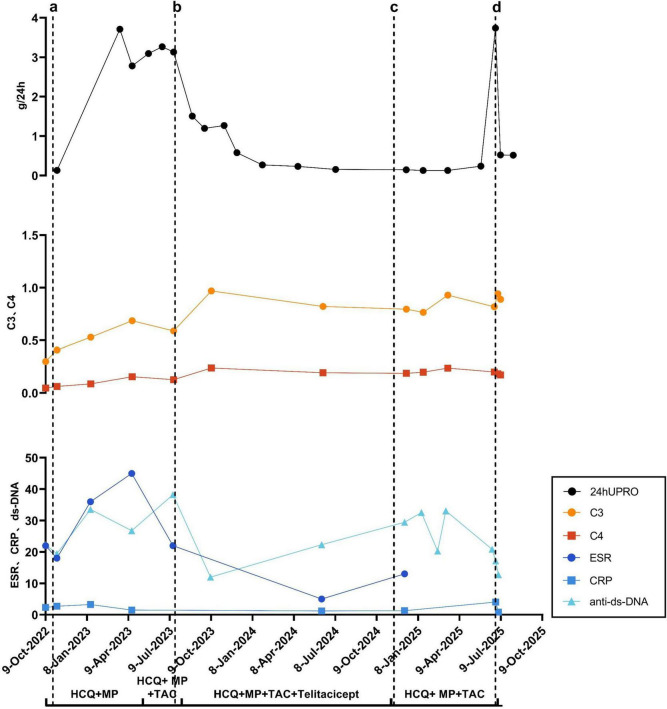
(a) First detection of proteinuria (positive); (b) Renal biopsy; (c) Last menstrual period; (d) Delivery of a physiologically normal infant. 24hUPRO, 24-hour urinary protein excretion (g/24h); C3, complement component 3 (g/L); C4, complement component 4 (g/L); ESR, erythrocyte sedimentation rate (mm/h); CRP, C-reactive protein (mg/L); anti-ds-DNA, anti-double-stranded DNA antibody (I U/mL); HCQ, hydroxychloroquine; MP, methylprednisolone; TAC, tacrolimus.

**FIGURE 2:**
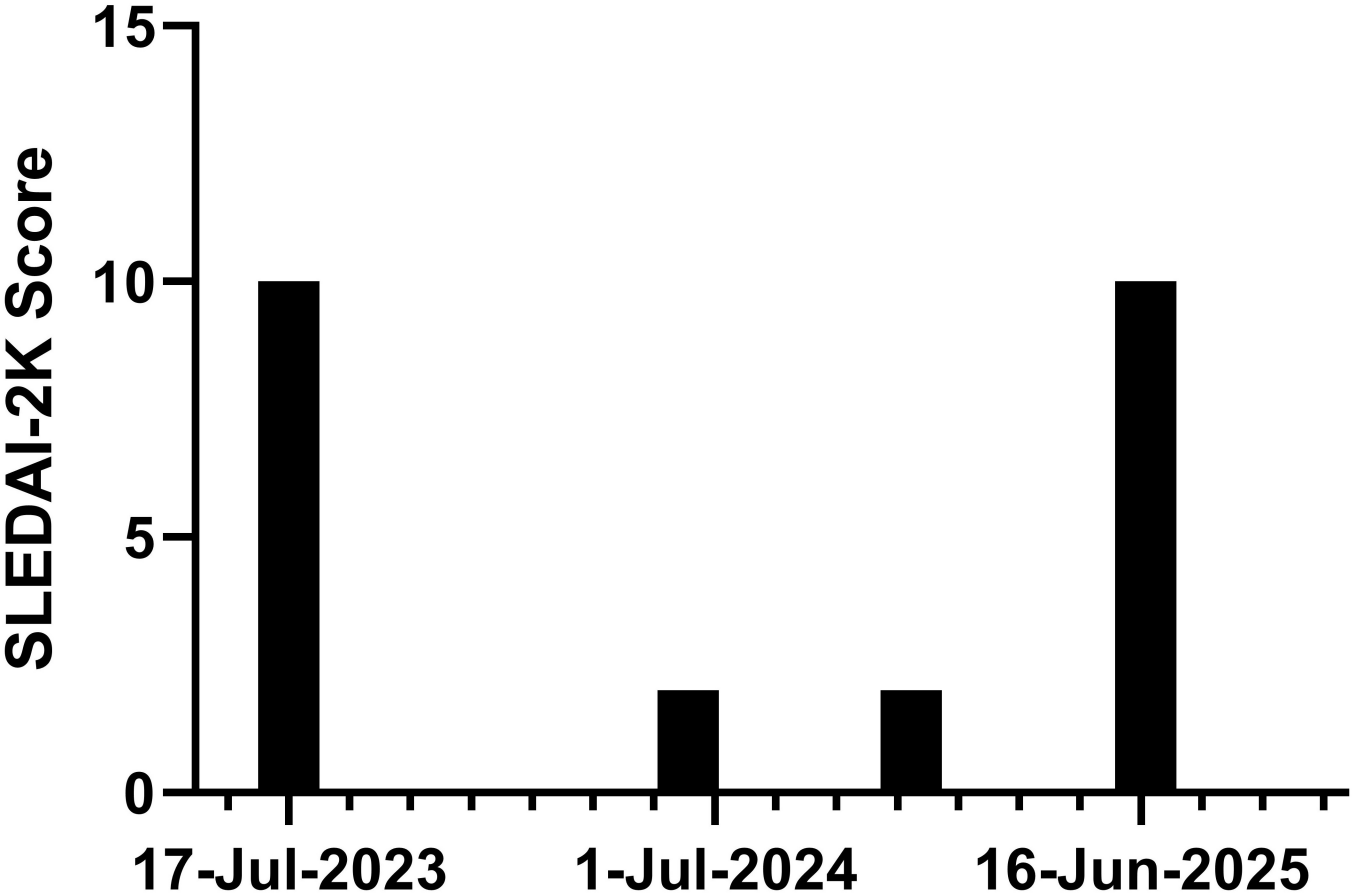
Timeline of SLE Disease Activity Index (SLEDAI-2K) scores. A score of 10 on 17 July 2023, indicated high disease activity and prompted a renal biopsy. Following treatment adjustment, the score decreased to two (indicating low disease activity) by 9 June 2024, and remained stable at this level through pregnancy confirmation on 9 December 2024. A subsequent flare occurred at 32 weeks of gestation (20 June 2025), with the score rising again to 10.

The patient was diagnosed with SLE in 2017 and initially managed at another hospital. She reported a prior induced abortion in 2020 due to a systemic lupus erythematosus flare that was clinically assessed as “highly active”; however, specific serological or organ involvement details from that episode are unavailable as the care was provided at another institution and contemporaneous records are not accessible. She had no other history of adverse pregnancy outcomes. The patient was diagnosed with systemic lupus erythematosus (SLE) at her first visit to our hospital in 2022 according to the 2019 EULAR/ACR classification criteria. The diagnosis was confirmed by a total score of 14 points, which included: a history of malar and hand rash consistent with subacute cutaneous lupus (4 points), a positive anti-dsDNA antibody test (6 points), and low levels of both complement C3 and C4 (4 points). A score of 10 or more is required for classification, which the patient met. No involvement of visceral organs was identified. The diagnosis was confirmed following the exclusion of infection, malignancy, and pharmacological causes. Since then, she has been regularly followed at our hospital and maintained on methylprednisolone and hydroxychloroquine, with low disease activity. Supportive care included vitamin D and calcium chewable tablet, alfacalcidol soft capsules and pantoprazole enteric-coated tablets. Throughout this time, the patient was unremarkable for any visceral organ involvement. Systemic review was negative for cutaneous, musculoskeletal, or mucocutaneous symptoms such as rash, arthralgia, alopecia, or oral ulcers. Foamy urine first occurred in December 2022. Urinalysis revealed a strongly positive result for urinary protein. The dosage was adjusted during the outpatient follow-up, but there was no significant improvement in proteinuria. In May 2023, a 24-h urinary protein quantification test revealed a result of 3.710 g/24 h. Oral tacrolimus was then added to the existing treatment regimen at 1 mg twice daily, which was subsequently increased to 1 mg once daily in the morning and 2 mg once daily at night with therapeutic drug monitoring confirming target trough levels. However, follow-up visits continued to show persistent proteinuria, and no significant reduction in 24-h urinary protein excretion was observed. The patient presented with fatigue and alopecia but denied other symptoms such as rash, oral ulcers, or arthralgia. The patient was then hospitalized in July 2023 for a renal biopsy, and was diagnosed with class V Lupus Nephritis. No involvement of other visceral organs was observed. The patient received the first treatment with telitacicept (160 mg, once weekly) on 31 July 2023, followed by regular injections combined with the aforementioned oral medications. Subsequently, her 24-h urinary protein quantification level decreased significantly compared with the previous level (Figure 1). In January 2024, the patient achieved low disease activity, and subsequent outpatient follow-up and re-evaluations showed stable disease status. We retrospectively evaluated the patient’s disease activity using the Systemic Lupus Erythematosus Disease Activity Index (SLEDAI-2K) (Figure 2). The score was 2 at pre-conception (9 June 2024), indicating stable, low disease activity. The patient’s last telitacicept injection was administered on 4 November 2024, and her last menstrual period (LMP) was on 7 November 2024. The score remained at 2 at pregnancy confirmation (9 December 2024). At this time, the only notable laboratory finding was a positive anti-dsDNA antibody. Other test results were within normal limits, and the patient reported no specific discomfort. Upon confirmation of pregnancy, telitacicept injection was discontinued, and the patient was treated with methylprednisolone, hydroxychloroquine, and tacrolimus (0.5 mg twice daily from January 2025 to April 2025, subsequently increased to 1 mg twice daily) with therapeutic drug monitoring confirming target trough levels. Aspirin enteric-coated tablets (75 mg, once daily at bedtime) were initiated in January 2025 following evaluation by an obstetrician. Subsequently, nadroparin calcium injection (0.4 ml, 4100 AXa IU) was administered subcutaneously once daily starting in February 2025, following assessment by an obstetrician. The rheumatologist, obstetrician-gynecologist, and the patient herself jointly formulated the treatment regimen during pregnancy. The patient had unremarkable prenatal visits at 17 and 24 weeks of gestation, with no signs of SLE flare-up. On 9 June 2025, at over 29 weeks of gestation, ultrasound examination revealed fetal growth restriction (FGR), with fetal size approximating 27 weeks. The obstetrician recommended hospitalization for comprehensive examinations and further evaluation, but the patient declined. On 20 June 2025, laboratory tests revealed elevated levels of total bile acids, aspartate aminotransferase, and alanine aminotransferase. A diagnosis of intrahepatic cholestasis of pregnancy (ICP) was established. Subsequently, the patient was admitted to the hospital following the recommendation of the obstetrician. After admission, the patient was found to have elevated blood pressure and was diagnosed with preeclampsia. Owing to the increased level of anti-double stranded DNA (anti-dsDNA), after consultation with the rheumatologist, it was considered that the lupus was active. At 20 June 2025, the SLEDAI-2K score reached 10. The laboratory workup was notable for strongly positive proteinuria, positive anti-dsDNA antibody, and the presence of cylindruria. Other test results were otherwise unremarkable, and the patient was asymptomatic. Based on the combined advice of the rheumatologist and obstetrician, the patient decided to terminate the pregnancy. On 23 June 2025, the patient underwent a cesarean section and delivered a male infant at a gestational age of 32^+4^ weeks. The infant had an Apgar score of 8 at 1, 9 at 5, and 9 at 10 min, with a birth weight of 1,490 g and no physical defects.

The male infant was admitted to the neonatology department due to “prematurity, very low birth weight, and neonatal respiratory distress syndrome.” Both ophthalmologic examination and newborn hearing screening yielded normal results. After a 21-day hospitalization, he was discharged in good general condition at a postmenstrual age of 36^+1^ weeks with a weight of 2.34 kg.

## 3 Discussion and conclusion

According to the study, the elimination half-life (t1/2) of free serum telitacicept is approximately 11.7 days, while that of conjugated telitacicept is prolonged, with a half-life of 19.4 days (10). In this case, telitacicept was discontinued 3 days prior to the last menstrual period. While the drug’s extended half-life suggests exposure likely persisted into the very early preconception period, our singular observation indicates that the impact of telitacicept on the preconception period appears to be minimal. The patient delivered an infant without physical defects at 32 weeks of gestation, which suggests that telitacicept did not result in structural defects in this case.

Regulatory T (Treg) cells play a critical role in early pregnancy by suppressing immune activation and maintaining maternal-fetal tolerance, thereby reducing the risk of pregnancy complications (11). However, SLE is often associated with dysregulation of immune cell subsets, including a decrease of Treg and T helper 2 (Th2) cells, along with an increase of T helper 1 (Th1) cells (12). Pro-inflammatory cytokines secreted by Th1 cells—such as interferon-gamma (IFN-γ), interleukin-12 (IL-12), and tumor necrosis factor-alpha (TNF-α)—may adversely affect fetal development in late gestation and contribute to pregnancy loss (13, 14). In contrast, Th2 cells produce anti-inflammatory cytokines including IL-4, IL-10, and IL-13, which promote B cell proliferation and differentiation. This leads to excessive autoantibody production by mature B cells and plasma cells, ultimately triggering inflammatory attacks on host tissues and organs. Consequently, pregnant individuals with SLE face higher risks of maternal complications such as preeclampsia, lupus flares, gestational diabetes, and perinatal mortality, as well as fetal adverse outcomes including preterm birth, intrauterine growth restriction, fetal loss, and congenital heart block compared to the general population (15, 16).

In this case, the patient did not experience miscarriage and successfully passed the prenatal examinations at 17 and 24 weeks of gestation. This outcome was the collective result of multiple pharmacological interventions. Apart from the use of telitacicept during early pregnancy, the medications administered throughout the pregnancy have established safety profiles supported by numerous clinical studies. Prospective studies indicate that exposure to maintenance doses of glucocorticoids during pregnancy does not significantly increase the risk of major fetal malformations (17). The safety of hydroxychloroquine for the fetus has been confirmed by extensive research, and its use is recommended in pregnant patients with systemic lupus erythematosus, as it not only reduces the risk of disease flare but also prolongs pregnancy (18–22). Tacrolimus is also recommended for use in pregnant lupus patients, with studies confirming no teratogenic risk from its use during pregnancy (3, 23, 24).

Beyond the protective effects of the aforementioned medications, the absence of miscarriage in this case might be attributed to the fact that telitacicept dual-inhibits the multi-stage development and differentiation of B lymphocytes by simultaneously blocking B lymphocyte stimulator (BLyS) and a proliferation-inducing ligand (APRIL) (25, 26). Whether telitacicept injection exerts a miscarriage-protective effect deserves further investigation.

At over 29 weeks of gestation, the patient was found to have FGR on prenatal ultrasound, with fetal size approximating 27 weeks. FGR is defined as failure of the fetus to achieve its growth potential, primarily caused by impaired development and dysfunction of the villous vascular system, leading to reduced placental blood flow (27). Several mechanisms may contribute to FGR in this setting. First, glucocorticoid use throughout pregnancy—as in this case—is a known risk factor for restricted fetal growth (15). Furthermore, patients with SLE often exhibit endothelial dysfunction. Activated platelet phenotype and type I interferon signal transduction together with high levels of soluble vascular cell adhesion molecule-1 (sVCAM-1) and endothelial microparticles (EMPs) are identified as important inducers of endothelial dysfunction and damage, which may adversely affect placental vascular development (12). Under physiological conditions, B cells help maintain immune tolerance, produce protective antibodies for the fetus, and support placental vascular growth. In SLE, however, autoreactive B cells generate autoantibodies (such as IgG) that can cross the placenta and cause damage to both the fetus and the placenta. Telitacicept may mitigate this process by inhibiting B cell proliferation. Nevertheless, whether telitacicept confers any protective effect on fetal growth remains to be investigated.

During hospitalization at 32 weeks of gestation, the patient was diagnosed with preeclampsia. The risk of preeclampsia in pregnant patients with SLE, especially those complicated with lupus nephritis, is higher than that in the general population. This may be due to the fact that SLE patients have an increased risk of arteriosclerosis caused by endothelial dysfunction (15). Further studies are needed to determine whether telitacicept increases the risk of preeclampsia.

In summary, this represents the first reported case of a patient with SLE exposed to telitacicept during early pregnancy who delivered an infant without any physical defects. This outcome suggests that telitacicept did not result in structural defects in this case and could potentially possess protective effects against pregnancy loss. However, the pharmacokinetic data and the precise drug concentration at the time of conception remain unknown in this case. Therefore, no causative conclusions or generalizable safety assurances can be made. Further clinical studies are warranted to validate these observations. The patient’s personal perspective and lived experience were not systematically captured in this retrospective report, which represents a limitation of the study.

The teratogenic risk of telitacicept has not been fully evaluated. All patients with childbearing potential must strictly use contraception during treatment with telitacicept and receive thorough pre-pregnancy counseling.

## Data Availability

The raw data supporting the conclusions of this article will be made available by the authors, without undue reservation.
